# Novel Virtual Reality Methods for the Treatment of Obsessive-Compulsive Disorder Using a User-Centered Design Approach: Focus Group Study

**DOI:** 10.2196/92410

**Published:** 2026-08-20

**Authors:** Michael Colman, Josie F A Millar, Bhagyashree Patil, Daniel J Finnegan, Mhairi Kristoffersen, Danae Stanton Fraser

**Affiliations:** 1 Department of Psychology, University of Bath, Claverton Down, Bath, England, BA2 7AY, United Kingdom, 44 7500750181; 2 Department of Computer Science, University of Bath, Bath, England, United Kingdom; 3 School of Computer Science and Informatics, Cardiff University, Cardiff, Wales, United Kingdom

**Keywords:** virtual reality, obsessive-compulsive disorder, user-centered design, participatory research, cognitive behavioral therapy, virtual reality exposure therapy

## Abstract

**Background:**

Obsessive-compulsive disorder (OCD) is a common mental health disorder that can cause significant impairment to occupational and social functioning. While effective treatments exist, outcomes are often hampered by issues outside of the treatment protocol. Virtual reality (VR) presents a potential solution to help support the effective administration of treatment, but its application in the field of OCD has thus far been limited to replicating real-world environments used in exposure exercises, despite the potential to generate environments and stimuli beyond what is possible in reality. Participatory design methods are an effective way to ensure that the development of novel interventions is relevant to the intended end users and is grounded in their expertise and experiences.

**Objective:**

The aim of the workshops conducted in this study was 2-fold. First, to understand the opinions of relevant stakeholders (clinicians and people with lived experience of OCD) on the current state of OCD treatment. Second, to generate novel ideas with the direct input of stakeholders for how VR might effectively be applied to support the treatment of OCD.

**Methods:**

In total, 6 clinicians with experience of treating OCD and 6 people with lived experience of OCD participated in a combination of round-table discussions in large groups, and creative design exercises conducted in smaller groups and individual interviews. All workshops and interviews were audio-recorded, transcribed, and analyzed using collaborative qualitative analysis. Themes were then synthesized via narrative synthesis.

**Results:**

Three themes relating to the first aim were identified: socializing to the model, effective exposure work, and therapeutic alliance (predominantly raised by people with lived experience of OCD). Five themes relating to the second aim were identified, with ideas for VR use covering 5 mechanisms by which it might support treatment: enabling effective exposure work, modeling, illustrating metaphors and the treatment framework, anxiety management, and motivation.

**Conclusions:**

Findings from the first workshops largely restate previous findings emphasizing the importance of clients developing a comprehensive shared understanding of the cognitive model of treatment with their therapist. People with lived experience of OCD also highlighted the importance of developing trust in their therapist’s expertise and ability to understand nuances of individual cases. Findings from the second workshop produced a wide variety of ideas for how VR could support the treatment of OCD. Many of these go beyond the concept of simply enabling exposure work as seen in previous literature. Methods to increase client engagement with the cognitive model during treatment were described, and people with lived experience proposed methods of managing anxiety experienced during treatment to facilitate approach behaviors. Avenues for further development of these ideas and areas for further refinement are discussed.

## Introduction

Obsessive-compulsive disorder (OCD) is a mental health disorder with an estimated global lifetime prevalence of 1%‐2% [1,2]. OCD is characterized by obsessions—recurrent and persistent intrusive thoughts, urges, and images that generate significant distress in the individual and are experienced as unwanted [3]. In response to these obsessions, the individual feels compelled to carry out compulsions—repetitive behaviors and/or mental actions intended to suppress or neutralize obsessions and/or reduce distress [3,4]. While the mechanisms remain consistent across cases, the content of obsessions and compulsions can cover diverse topics, from concerns about physical contamination to immoral or socially taboo thoughts [5,6]. OCD can significantly impact individuals’ social and occupational functioning [7], and approximately 14%‐26% of people with OCD report attempting suicide at least once in their lifetime [8,9].

Given the heterogeneity of symptoms and other issues, such as common comorbidities, OCD can be a complex disorder to treat [10]. Psychotherapeutic treatment in the form of cognitive behavioral therapy (CBT) with exposure and response prevention (ERP) is widely considered an effective treatment for OCD [11,12]. However, there is also consistent evidence that a significant proportion of people with OCD do not receive CBT, or if they do, it is not technically adequate. For example, a systematic review examining patient adherence to CBT found that 15% (n=877) of eligible patients do not take up CBT at all, while a further 15% (n=139) drop out of treatment [13]. Even when patients do choose to engage in a course of CBT, studies suggest that the inclusion of ERP (a key active component of CBT) varies between 16% and 50% [14,15]. Additionally, when ERP is included, it is sometimes administered incorrectly or inconsistently. For example, the use of imaginal exposure (where the patient is required to imagine facing their feared stimuli) rather than in vivo (ie, where the patient is physically present in facing their fears) [16,17]. In some situations, imaginal exposure has been shown to be less effective than in vivo exposure, as patients might not engage as much with imaginal stimuli and can use cognitive strategies to avoid confronting situations in a manner conducive to effective CBT [18]. As such, there is increasing interest in developing methods to support the effective delivery of treatment for OCD.

Virtual reality (VR) technology has been suggested as one such method. The term “VR” refers to a viewer-centered experience in which the user feels as if they are present in a simulated 3D virtual environment (VE) separate from their physical environment [19,20]. Technologies such as head-mounted displays are used to achieve the sensation of seeing and looking around in a VE, while input devices such as controllers can be used to allow people to navigate and interact with the VE. With exposure work being such a critical part of CBT, VR can provide exposure situations more impactful than imaginal, but without the practical difficulty of in vivo [21]. For those people whose struggles with OCD prevent them from leaving their homes or have comorbid conditions such as depression, VR can also be a tool that enables access to exposure environments that are relevant but currently unrealistic to attempt [22]. Additionally, the level of control VR affords means exposure scenarios can be personalized to be relevant to individual patients’ particular difficulties [23], which is pertinent given the emphasis people with OCD have placed on the need for personalized therapy materials [24].

There is substantial evidence that VR can evoke the necessary anxiety to be an effective tool in exposure work with OCD, but the research surrounding its ability to provide long-term treatment outcomes is comparatively underdeveloped [25]. Another important feature of the extant literature around VR and OCD is that all apps developed and tested thus far only add value to current treatment protocols by making exposure environments more accessible and approachable. VR enables the generation of realities with elements and environments that go beyond what is possible in the real world [20]. As such, there is scope to investigate how this capacity might be harnessed to add value to treatment in novel and previously unconsidered ways [26]. However, it remains unclear how best to implement VR effectively within OCD treatment, highlighting the need for the development of novel VR methods to support clinical practice.

Participatory design methods are increasingly being applied to the development of VR apps in therapeutic contexts and may help bridge this gap between conceptual innovation and clinical implementation [27,28]. More broadly, there is a growing call for all therapeutic interventions for OCD to be codeveloped as best practice [29]. The person-based approach (PBA) to intervention development posits that consistently involving end users and relevant stakeholders in the iterative design and development of interventions produces the best development results, maximizing both acceptability and effectiveness [30]. This involvement enables researchers to develop a nuanced understanding of the intended users’ experiences, which in turn informs intervention design. Concurrently, it allows intended users to directly contribute to the core content of the interventions to ensure their relevance and impact [31]. The current Medical Research Council (MRC) guidance for complex intervention development highlights the importance of stakeholder involvement during the development phase of new interventions [32]. This study aimed to generate novel ideas for how VR could be used to support or augment the treatment of OCD through participatory design methods. In line with the PBA [30], we aimed to ground these ideas in an understanding of the relevant stakeholders’ psychosocial context, including past experiences of treatment, motivations to engage in interventions, and perceived barriers to effective treatment. To achieve these goals, the study adopted a constructivist interpretive approach where stakeholder accounts were used to generate contextualized, actionable design ideas in an exploratory way. The inductive approach of the collaborative qualitative analysis was chosen in line with this constructivist approach. To do this, 2 research questions (RQs) were developed:

1. How do clinicians and people with lived experience of OCD feel about the current state of OCD treatment? (ie, what do they perceive works effectively and what could change?)
2. How do clinicians and people with lived experience of OCD envision using VR in the treatment of OCD?

## Methods

### Overview

This study has been reported in line with the Journal Article Reporting Standards for Qualitative Primary Research in Psychology (JARS-Qual) [33].

### Participants

Two stakeholder groups independently took part in the study: clinical professionals with experience of treating OCD (hereafter referred to as clinicians) and people with lived experience of OCD. Clinicians and people with lived experience of OCD did not participate in the same workshops; all workshops involved only one stakeholder group. However, ideas generated in one group were sometimes shared with the other by the researchers as part of workshop discussions.

Clinicians were recruited through the clinical supervisor’s contact network and snowball sampling methods. Following initial participation in Workshop 1, some participants suggested that the research team contact colleagues who might be interested in participating. In total, 6 different clinicians took part in Workshop 1, Workshop 2, or both. They were aged 39‐53 (mean 47.33, SD 4.84) years, and 4 out of 6 were female. Professional background and experience data were not collected for all participating clinicians due to incomplete demographic forms; however, the inclusion criteria for the study meant that all participating clinicians had more than 10 years’ experience of providing CBT treatment to people with OCD.

People with lived experience of OCD were recruited from a group of people with OCD who had expressed their interest in participatory design and taking part in OCD research to the research team. Some of these people had worked together in previous research projects. These people had attended conferences related to OCD and requested to be contacted by the research team if research opportunities arose. All participants recruited in this study had undertaken a course of CBT for OCD and were at a stage in their recovery where they felt psychologically safe to engage in group discussions about OCD and treatment. In total, 6 people with lived experience of OCD took part in one or both of Workshop 1 and/or Workshop 2. They were aged 34‐50 (mean 42, SD 6.36) years, and 4 out of 6 were female. Data regarding treatment history were not collected for all participants due to incomplete demographic forms (Table 1).

**Table 1.** Demographic characteristics of Workshop 1 and 2 participants.

Characteristic	Clinicians (n=6)	People with lived experience of OCD^a^ (n=6)
Sex (female), n (%)	4 (66.67)	4 (66.67)
Age (years), mean (SD)	47.33 (4.84)	42 (6.36)
Ethnicity, n (%)		
White British^b^	6 (100)	6 (100)
Treatment background, n (%)		
Clinical psychologist	3 (50)	—^c^
CBT^d^ therapist	1 (16.67)	—
N/R^e^	2 (33.33)	—
Treatment experience in years, mean (SD)	18.75 (6.29) (N/R= 2)	—
Age when first seeking treatment for OCD (years), mean (SD)	—	23 (9.31) (N/R=2)

a OCD: obsessive-compulsive disorder.

b Participants described their own ethnicity rather than selecting a preset option.

c Not applicable (used when demographic information applied only to one stakeholder group).

d CBT: cognitive behavioral therapy.

e N/R: not reported (used when participant demographic data were missing).

A potential concern is that familiarity with the clinical supervisor and/or each other might bias participants toward endorsing each other’s or the researchers’ views and opinions, rather than expressing their own. However, the topics being discussed and participatory design methods used were different from any of the participants’ previous experiences of taking part in OCD-related research. An important part of the process was authentic reflection on the topic at hand, and it was considered that these potential biases were unlikely to impede such reflection. Moreover, engaging with an already established group of stakeholders offered the advantage of a greater level of psychological safety. This is important for encouraging honest reflection and confidence to engage in the creative activities necessary to the methods of the workshops. Additionally, having already worked together as a group on different projects, they had established that they were comfortable working collaboratively in the participatory design atmosphere of the study.

Safety measures were kept in place and made clear to participants throughout the workshops, including the option to mute, turn off the camera, or leave the Microsoft Teams meeting during workshops, or withdraw from the study between workshops or at any time during a workshop. In all workshops with people with lived experience of OCD, the lead supervisor, a clinical psychologist, was present throughout to provide psychological support and/or to offer follow-up contact if needed.

### Ethical Considerations

Ethical approval for conducting the study was obtained from the University of Bath Psychology Research Ethics Committee (Application Number 22‐107). When participants were first approached about participating, they were presented with an information sheet detailing: the purpose and procedures of the study and how the data they provided would be used for intended research outputs. They were informed of their right to withdraw their participation or data from the study either during or between workshop sessions, with the caveat that the data they contributed during group workshops could not be separated from the wider group due to the audio recording and transcription process. Once participants had read this information, they were presented with an informed consent form to sign if they wished to participate. The details of the Bath Psychology Research Ethics Committee were also provided if they had any concerns or queries. Participant contact details were kept confidential by storing them on the University of Bath’s secure network. Once audio data had been recorded and transcribed, any personally identifying data were removed from the transcripts; participants were assigned a code identifier to differentiate contributions, and their contact details and names were deleted for anonymity. Participants were compensated for their time at a rate of £20 (US $26.81) per hour in line with best practice guidance [34].

### Design

Two different workshop designs were implemented to address the RQs, hereafter referred to as Workshop 1 and Workshop 2. In total, 8 workshops were conducted: 2 Workshop 1s, followed by 6 Workshop 2s. Each workshop used different methodologies tailored to the stage of the participatory design process [35].

For Workshop 1, a focus group method involving open discussions was used to explore RQ1. The focus group method was appropriate for answering RQ1 as it enabled the collection of rich information on complex topics, while allowing clarification and elaboration as participants built on each other’s contributions [36,37]. The aim of Workshop 1 was to provide researchers with a clear understanding of the context of current OCD treatment and to provide contextual scaffolding to inform the conduct of Workshop 2.

Workshop 2 used several creative design methods, including design games or “icebreakers” and the persona-and-scenario exercise [38] to generate ideas for methods to improve or augment OCD treatment, addressing RQ2. These methods are designed to enhance participant creativity by encouraging open exploration unconstrained by practical limitations [39]. This makes them ideal to promote the generation and exploration of novel applications of VR in OCD treatment. The aim of Workshop 2 was to generate novel ideas for how VR could be applied to support the treatment of OCD. As such, the intended output of the study was an analysis of the discourse from the workshops capturing the range of ideas produced along with participants’ clarifications and elaborations (refer to the “Results” section).

Both workshops were conducted online via Microsoft Teams, facilitated by members of the research team. The research team consisted of the lead researcher, the clinical supervisor (a clinical psychologist), and a research assistant.

### Procedure

#### Overview

Participants were initially approached by the clinical supervisor through their professional network and provided with an online information sheet and informed consent form, which included contact details for any questions. After returning the form, participants were contacted by the lead researcher to organize a time to meet.

#### Workshop 1: Focus Group Discussions

Two Workshop 1 sessions were conducted: one with people with lived experience of OCD and one with clinicians. The people with lived experience of OCD focus group lasted 2 hours and included 5 participants, while the clinicians’ focus group lasted 90 minutes and included 4 participants. At the start of each session, the lead researcher introduced the research team and the intended purpose and outputs of the workshop. Stakeholder participants then introduced themselves, with this opening segment taking approximately 15 minutes. This was followed by 2 open whole group discussions, separated by a short break.

Discussions were guided by 2 text prompts: “past experiences of receiving/providing treatment for OCD*”* and “improving the treatment of OCD.” Where needed, the lead researcher drew on a preprepared list of questions related to each prompt to stimulate further discussion or clarify participants’ views (Multimedia Appendix 1). The first discussion explored participants’ perspectives on aspects of treatment they felt worked well, as well as features that they perceived were less effective or overlooked. The second discussion prompt was “improving treatment.” In a similar method to the first discussion point, this prompt was focused on highlighting areas of the current treatment protocol which could be improved or receive more attention, as well as introducing ideas outside of the protocol itself. Each discussion lasted approximately 45 minutes for people with lived experience of OCD and 30 minutes for clinicians. The clinical supervisor was present throughout both Workshop 1 sessions, fulfilling slightly different roles in each stakeholder group. In both groups, they helped mediate discussion and provide clarifications between the participants and lead researcher. In the people with lived experience of OCD group, the supervisor also acted as safeguarding lead, monitoring discussions to ensure that participants were not triggered by the content.

Participants were encouraged to contribute using the “raise hand” feature or by typing in the chat. To further support collaboration, a brainstorming platform (Padlet [Nitesh Goel]) was shared with participants and displayed throughout the session. Participants added their ideas to the Padlet board via virtual “post-its.” The research assistant monitored and organized the board, recording verbal contributions and editing entries to maintain legibility and structure for the group discussions.

Following the discussion, the lead researcher delivered a brief educational presentation (approximately 10 minutes) on VR, its applications to OCD thus far, and other mental health problems, and examples of novel therapeutic uses (Multimedia Appendix 2). The first example was Oxford VR’s acrophobia treatment app, which uses gamification and an automated therapist agent to treat fear of heights [40]. The second illustrated a “feedforward” mechanism, used in athletic training, in which a cyclist’s recorded performance is augmented and represented by an in-VR agent that the cyclist then races [41]. The purpose of the presentation was to broaden participants’ thinking, emphasizing VR’s capacity not only to replicate reality but also to generate new realities with features beyond what was possible in real life. Participants were invited to reflect on this capacity in advance of Workshop 2. They were asked to consider how VR might change or generate realities rather than focusing on technological constraints, as the next workshop prioritized idea generation over technical refinement.

Afterward, participants were thanked and offered a debrief exercise to help facilitate the transition back into their regular day. Following session closure, the lead researcher contacted participants via email to schedule Workshop 2.

#### Workshop 2: Creative Design Exercises

Due to the nature of the exercises and the difficulty of rescheduling all participants to meet at the same time, 6 Workshop 2 sessions were conducted. Clinician workshops were run with 1 or 2 participants and lasted 1 hour to accommodate scheduling constraints. People with lived experience of OCD workshops were held with at least 2 participants and lasted 2 hours, with the clinical supervisor present throughout. For clinicians, it was considered safe to participate individually, as their contributions were based on synthesizing professional experience rather than reflecting on personal experiences. By contrast, people with lived experience of OCD workshops involved greater personal reflection, so these sessions required at least 2 participants and the presence of the clinical supervisor to ensure safeguarding.

The workshop structure differed slightly across stakeholder groups, given clinicians’ shorter sessions. Both groups began with a brief summary of the discussions and output of Workshop 1 (ie, a list of key treatment features and areas to improve). For participants attending only Workshop 2, a short presentation on VR and its potential use in treatment was also provided. This introductory process lasted approximately 10 minutes in both groups.

People with lived experience of OCD workshops began with an icebreaker and warm-up design game called “Crazy 8’s” or “8-Box Blitz” [42]. Participants were asked to respond to a broad prompt (“ways to make OCD treatment better”) by filling 8 boxes on a sheet of paper with ideas under a short time limit. The exercise aimed to encourage increasingly unorthodox thinking without allowing time for refinement or consideration of feasibility. Ideas were briefly shared and discussed among participants and researchers. In both people with lived experience of OCD and clinician workshops, participants then used a worksheet (Multimedia Appendix 3) and discussion to create “personas”—characters representing people seeking treatment for OCD, the target audience for the VR intervention. The research assistant took primary responsibility for recording participant contributions on the worksheet, while the lead researcher facilitated deeper persona development by asking clarifying questions.

Following a break, the personas were used as the basis for a scenario exercise. Participants were asked to describe what their persona’s treatment might ideally look like in a world where VR technology was more advanced and widely available. A Padlet board with a timeline structure was displayed to support storyboarding of these scenarios. While the structure provided scaffolding to guide idea generation, participants were encouraged to share thoughts and opinions in a free-flowing manner.

In both workshops, once the persona and scenario exercises were completed, the lead researcher summarized the ideas generated during the workshop. Participants were informed that their data would be analyzed and the results would be prepared for publication, which they would be able to access. They were told that one idea from the workshops would be selected for further development, and that the lead researcher might contact them to provide feedback on the prototype. Finally, participants were offered an optional debrief exercise to help transition back into their regular day.

### Analysis

All workshops were audio recorded and transcribed. Transcripts were analyzed using collaborative qualitative analysis (CQA) [43]. This 6-phase method, developed by Richards and Hemphill, is broadly rooted in thematic analysis but uses structural elements of the constant comparative method to balance rigor, trustworthiness, and transparency while addressing the challenges of analyzing qualitative data in a team environment. The decision was made to use this approach with multiple analysts due to its structural support for developing qualitative researchers and its integration of the perspectives provided by multiple researchers. In this instance, the team responsible for analysis (henceforth called “the research team”) consisted of the lead researcher, a research assistant, and a clinical supervisor.

In phase 1, the research team met to discuss the analysis plan, including a week-by-week plan of team meetings, the data analysis plan, and coding assignments. The lead researcher and research assistant acted as primary and secondary coders, while the clinical supervisor acted as peer debriefer. At this stage, the research team also decided to take an inductive “data-driven” approach, which did not base the analysis on a preexisting theoretical framework. This was due to the exploratory nature of the study and its focus on ideation. However, it was noted that the analysis may often be informed by the cognitive approach to OCD because the majority of the participants’ experiences and opinions were rooted in the cognitive behavioral treatment model.

In phase 2, both coders independently and iteratively open- and axial-coded 3 of the 8 transcripts (38% of the data) over several rounds. Coders used Microsoft Word’s comment function for this stage of open-coding, additionally independently writing memos after each round of coding and meeting regularly to discuss relationships between codes and discuss unclear cases. Phase 3 was initiated once the coders both felt comfortable that they were regularly identifying a variety of patterns between codes. The primary coder reviewed meeting notes, memos, and coding of both coders to develop a preliminary codebook of themes and subthemes. For this process, the qualitative data analysis software LiGRE (Logiciels Ex-l-Tec Inc) was used so that themes and subthemes could be adjusted iteratively as necessary for all coders. The preliminary codebook was discussed in a meeting and given to the peer debriefer to review and suggest adjustments. When this was complete, the preliminary codebook was pilot-tested in phase 4 by having the 2 coders apply it to previously uncoded transcripts. Discrepancies and questions about the structure of the codebook were iteratively discussed in meetings between the coders, with modifications being made as necessary and then applied to new transcripts again by both coders independently. When developing the codebook regarding how stakeholders felt VR could be applied to the treatment of OCD, a decision was made to separate specific ideas into subthemes, while the proposed mechanism by which ideas might support treatment was used to distinguish themes. When both coders felt confident about the codebook, the peer debriefer reviewed it again. In phase 5, the coders divided the remaining transcripts and independently coded them according to the updated version of the codebook. Although split coding was used, meetings between coders were still conducted regularly to discuss excerpts where coders felt unsure. A final review was then conducted where both coders reviewed all coded transcripts to check consistency, resolve any uncertainties or disagreements between coders, and decide how to handle outliers. Finally, in phase 6, the research team developed the codebook into a thematic structure to describe the perspectives of the participants, which can be used in the write-up of the analysis.

Following this, the lead researcher produced a summary of themes from Workshop 1 and a narrative synthesis of themes identified in the data from Workshop 2. From Workshop 1, 3 major themes were developed: (1) the importance of developing a shared understanding of the cognitive treatment model for OCD with clients, (2) the importance of high-quality and balanced exposure work and behavioral experiments, and (3) the importance of a strong therapeutic alliance between the client and clinicians. For Workshop 2 which focused on novel applications of VR to the treatment of OCD, 5 major themes were developed: (1) VR to support or in some cases enable effective exposure work, (2) VR as a modeling tool, (3) VR to visually illustrate aspects of the treatment model to clients, (4) VR as an “anxiety management” tool, and (5) VR as a motivational tool. For a full table of themes, subthemes, and codes from each stakeholder group, refer to Tables 2 and 3. The results were organized by RQs, themes, and subthemes, rather than by workshops or stakeholder group. This was because, while the workshops had distinct aims, themes relevant for both RQs were identified across both workshops and from both stakeholder groups.

**Table 2.** Themes, subthemes, and code data for research question 1^a^.

Themes and subthemes	Codes
Socializing to the model	
—^b^	24 codes 3 clinicians (19) 3 people with lived experience of OCD^c^ (5)
Exposure work and behavioral experiments	
Effective timing of exposure work and behavioral experiments	17 codes 3 clinicians (8) 3 people with lived experience of OCD (9)
Keeping the quality of work consistent and model-focused	16 codes 2 clinicians (8) 4 people with lived experience of OCD (8)
Therapeutic alliance	
Establishing and maintaining a compassionate stance	18 codes 2 clinicians (5) 4 people with lived experience of OCD (13)
The therapist’s theoretical understanding of OCD	17 codes 6 people with lived experience of OCD (17)
The therapist’s ability to understand nuances of an individual case	20 codes 1 clinician (1) 5 people with lived experience of OCD (19)

a For each subtheme, the total number of codes identified is recorded. Then, the number of members of each stakeholder group who mentioned these codes is recorded. Finally, the number of codes coming from each stakeholder group is also recorded next to them (in brackets).

b Not available.

c OCD: obsessive-compulsive disorder.

**Table 3.** Themes, subthemes, and code data for research question 2^a^.

Themes and subthemes	Codes
Supporting and enabling effective exposure work	
Enabling exposure for impossible or imaginal scenarios	27 codes 4 clinicians (20) 2 people with lived experience of OCD^b^ (7)
VR^c^ as a “stepping stone” for in vivo exposure and supporting work outside the office	19 codes 5 clinicians (12) 4 people with lived experience of OCD (7)
Modeling behavior and experience	
—^d^	12 codes 4 clinicians (6) 2 people with lived experience of OCD (6)
Illustrating metaphors and the treatment framework	
Representing an externalized OCD	33 codes 3 clinicians (26) 3 people with lived experience of OCD (7)
Keeping the treatment framework present	28 codes 5 clinicians (24) 3 people with lived experience of OCD (4)
Anxiety management	
When overwhelmed during exposure	18 codes 2 clinicians (2) 4 people with lived experience of OCD (16)
For grounding purposes outside exposure	20 codes 1 clinician (5) 4 people with lived experience of OCD (15)
Motivation	
—	36 codes 4 clinicians (19) 2 people with lived experience of OCD (17)

a For each subtheme, the total number of codes identified is recorded. Then, the number of members of each stakeholder group who mentioned these codes is recorded. Finally, the number of codes coming from each stakeholder group is also recorded next to them (in brackets).

b OCD: obsessive-compulsive disorder.

c VR: virtual reality.

d Not available.

### Reflexivity

The research team consisted of the lead researcher (a PhD student researching OCD and VR), a research assistant (Master’s graduate in applied clinical psychology), and the clinical supervisor (a clinical psychologist specializing in CBT for OCD). The lead researcher had experience working within cross-disciplinary VR research but no prior clinical experience of OCD and no previous experience conducting coproduction research. The research assistant had previous experience conducting OCD-related research, including studies using coproduction methods and the PBA. The clinical supervisor brought 18 years of clinical experience treating OCD and previous research experience developing OCD interventions using coproduction methods.

The lead researcher conducted the workshops and acted as the primary coder and author of the analysis; the research assistant supported the conduct of the workshops and acted as the second coder; and the clinical supervisor provided pastoral support during workshops with people with lived experience of OCD and acted as the peer debriefer during analysis. While the clinical supervisor was present during these workshops, they did not actively contribute to idea generation and only acted in a safeguarding capacity. During the analysis procedure, the clinical supervisor also did not make any unilateral coding decisions, but provided clarification on clinical terminology and frameworks to the coders.

The study was informed by the PBA and conducted by a team with interests in OCD treatment, intervention development, and VR. As such, the authors acknowledge that participants’ accounts were interpreted through clinical, technological, and intervention-development perspectives, which may have influenced the construction of themes. The clinical supervisor had existing professional relationships with most participants, which may have influenced what participants felt comfortable sharing, although these relationships may also have facilitated trust and psychological safety during workshops. To promote reflexivity throughout the study, both coders maintained reflexive journals, recorded analytic memos, and met regularly to discuss assumptions and interpretations.

## Results

### RQ1: How Do Clinicians and People With Lived Experience Feel About the Current State of OCD Treatment?

Three themes were developed that represent how both clinicians and people with lived experience of OCD perceived the current state of psychological treatment for OCD and its most important features to provide scaffolding for idea generation in Workshop 2. These three themes are (1) developing a shared understanding of the model; (2) quality exposure work and behavioral experiments, which comprises 2 subthemes (effective timing of different exposure exercises and keeping the quality of exposure work consistent and model-focused); and (3) therapeutic alliance, which comprises 3 subthemes (establishing and maintaining a compassionate stance; the importance of the therapist’s theoretical understanding of OCD; and the importance of the therapist’s ability to understand individual patients’ cases). The importance of these 3 themes is largely well-established in the literature surrounding CBT for OCD [44-46], but they are briefly summarized here to contextualize the findings from Workshop 2.

Both stakeholder groups appreciated the importance of therapists and their clients properly understanding and applying the cognitive model to all aspects of treatment. This goes beyond the therapist understanding the model and ensuring it is applied to also ensuring the client has understood and “bought in” to the model, its necessity, and the effort it takes.

Getting the buy-in is absolutely crucial, so how you set it up, the understanding, by doing your Theory A, Theory B, so by the time you’re doing the active elements, which are absolutely crucial […] they've already bought into the need to do that.

[C2]

Similarly, both stakeholder groups felt that effective exposure work was a key element of the success of treatment. They emphasized the importance of keeping the exposure work’s quality consistent and focused on the cognitive model. This was raised as an area of difficulty by both groups, highlighting that therapists are not always able to regularly check in on their clients to support the treatment work they do outside the office and “squeeze the learning out of it” (C1). Effective exposure work was also characterized by the stakeholders as being a balance between scheduled and unscheduled exercises, and clinicians and people with lived experience of OCD highlighted that exercises based on an adaptable plan allowed for the client to move at a pace that best allowed for applying learning and growing confidence. However, the benefit of unplanned exercises was also highlighted, with both groups suggesting these might be more effective at dismantling OCD beliefs due to their unexpected and uncertain nature.

When I knew what was coming, it was easier, if that’s even the right word, than what I didn’t know was coming, and those were the best ones for me to get better.

[P4]

Finally, both groups highlighted the importance of a strong therapeutic alliance between client and clinician, although people with lived experience of OCD spoke more on this topic and suggested a different area to improve than clinicians. Both groups highlighted the importance of maintaining a compassionate stance to establish trust that the therapist will support clients to engage fully with treatment. Additionally, people with lived experience of OCD highlighted how the therapist’s demeanor can be critical in allowing clients to fully explain the breadth and nature of their concerns, particularly concerning taboo obsessions.

People don’t always admit everything that they perhaps might admit- that more sort of perhaps less taboo aspects of OCD […] and they’ll leave some of it out that they don’t wanna talk about. So I guess it’s all about getting, making the person, the patient, feel comfortable enough to be okay with doing that.

[P5]

Otherwise, exclusively people with lived experience of OCD highlighted how variance in the quality of treatment in their experience was often caused by variance in the therapist’s expertise and ability in 2 areas: their theoretical understanding of OCD and their understanding of an individual’s case. Strong theoretical understanding of OCD was said to increase clients’ confidence in their therapist’s ability to help them, and several people with lived experience of OCD recounted negative experiences of treatment stemming from a lack of theoretical understanding. The therapist’s understanding of a patient’s idiosyncrasies was an often-reported problem by people with lived experience of OCD, who felt that this understanding allowed for greater adaptability of the CBT protocol. This adaptability was highlighted as an area for improvement and was, in turn, thought to help motivate clients to engage with therapy and help them navigate situations where they felt the CBT protocol was not working for them.

### RQ2: How Do Clinicians and People With Lived Experience of OCD Envision Using VR in the Treatment of OCD?

#### Overview

Five themes were developed encompassing the ideas clinicians and people with lived experience of OCD generated regarding how to apply VR to the treatment of OCD: (1) supporting and enabling effective exposure work, (2) modeling typical behavior and “self”-modeling, (3) illustrating metaphors and the treatment model, (4) “anxiety management,” and (5) motivation. Themes 1, 3, and 4 each have 2 subthemes. These themes were not separated based on stakeholder group, as several were independently coded in both groups. Instead, to best examine differences or similarities of perspective, data relating to each theme were grouped together (with specific references to stakeholder group [clinician=Cx; people with lived experience of OCD=Px] noted where relevant).

#### Theme 1: Supporting and Enabling Effective Exposure Work

##### Overview

This theme highlights how clinicians and people with lived experience of OCD felt VR could benefit treatment simply by increasing the possibility and accessibility of exposure work for clients. The ideas contained in this theme extend upon existing literature suggesting VR can increase access to exposure scenarios by demonstrating OCD-specific situations which could be especially well-addressed by the reality generation capabilities of VR. The ways in which VR could be applied for this purpose are delineated in 2 subthemes.

##### Enabling Exposure for Impossible or Imaginal Scenarios

Clinicians suggested that an obvious use for VR’s reality-generation capabilities is as an effective replacement for imaginal exposure. Imaginal exposure is an exposure exercise where the client uses mental imagery to expose themselves to anxiogenic situations in their imagination. Clinicians highlighted that some cases can be extremely difficult to treat with effective in vivo (in-person) exposure work, particularly with symptom dimensions of OCD where the necessary exposure scenarios are not possible to facilitate.

Clinicians proposed 2 types of impossible scenarios that could be enabled through VR. First, scenarios that are practically too difficult to facilitate, for example, going to a busy swimming pool with children around (to address obsessions related to pedophilia). Second, scenarios that are physically impossible to facilitate, such as causing harm to come to loved ones or moral obsessions about the afterlife. In both types of scenarios, clinicians proposed that VR would essentially act as a more visceral replacement for imaginal exposure, creating impactful scenarios in instances where in vivo is not possible, thus enabling effective exposure work with clients.

I tried just the stunt walking on the plank thing, and […] rationally, you know that you’re on the floor, but it doesn’t feel it and I think that is the powerful thing about VR is that it does capture the emotion in a way that I think most rational people wouldn’t think is possible and in physical sensations, you know, get right into that. Even though I can say ‘I know I’m on the floor, I can walk, whichever,’ I know that it’s still hard to do. So, there is something about it which is more powerful than imaginal exposure, I think, and I’m slightly reluctant to say that, but...

[C6]

There was debate, from both clinicians and people with lived experience of OCD, over how far this concept of generating impossible or nonreal scenarios could be usefully implemented. For example, the ideas of a “worst case scenario” (C3) exposure or visually demonstrating the spread of contamination as imagined by a person with OCD in an exposure exercise were suggested. Arguably, both could be useful exposures: the former allows clients to challenge beliefs related to coping with feared outcomes. The latter increases the intensity of the exposure, potentially making the work done during it more impactful (“I would want to face absolute destruction and know that I could still go in and make dinner*”* P7). However, both ideas received criticism: learning to tolerate doubt and uncertainty of potential outcomes is an important part of treatment. Concerns were raised that using VR to show something that you normally cannot see (such as the spread of germs) might do more harm than good.

I’m not sure if that- in a way I’m, I just think perhaps it, that would feed into someone’s OCD in that you’re kind of, you know, making it visual, something that- that wasn’t before? Umm, that’s just my personal kind of thing, I don’t know, I just feel like that might be utterly terrifying, and like worse than reality in some ways.

[P5]

A further benefit raised by clinicians and people with lived experience of OCD was the potential for increased personalization of exercises to the client. It was felt that personalization would increase ecological validity and impact of exposure work, allow for transferring learning to different relevant environments, and increase motivation as client goals could be worked into the exercises. Both groups acknowledged the difficulty of balancing between widely applicable VR apps and the idiosyncrasy of OCD presentations.

I mean, you could model the place as well, couldn’t you, so then you‘re obviously changing the location, so she could have an office, lunch hall, she could have her dad’s house so that the stuff that she’s learning can be applied to different areas as well.

[P7]

##### VR as a “Stepping Stone” for In Vivo Exposure and Supporting Work Outside the Office

Clinicians and people with lived experience of OCD consistently felt that VR, through its ability to induce a sense of presence, could support treatment as part of a hierarchy as an introductory step toward in vivo exposure. Both groups mentioned examples of people being “stuck” when it came to exposure work and felt that this “stepping stone” use of VR could be the answer in a lot of cases.

For me the kind of beauty of VR would be on the one hand, my brain is absolutely in it. I’ve touched it. I’ve done this. I’ve done that. I’ve got that whole feeling. But there’s a little part of my brain can go ‘OK, I can try these things out. I can try and tolerate the anxiety I’m feeling’ without having that whole fear of that undoing.

[P1]

Within the bounds of the scenario exercise (a future where VR is more commonplace and available than it is today), clinicians also felt VR could be used to support exposure work done outside the office, for example, through remote work or homework. They proposed the control VR allows over the environment could ensure exposure exercises were run in a consistent manner, whether the therapist was present or not. For example, clients could do effective work in a session, the therapist could note what worked best and then provide a structured recreation of that via VR to be taken away for homework. This version of homework would remove extraneous variables that might affect client homework otherwise.

### Theme 2: Modeling Typical Behavior and “Self”-Modeling

This theme focuses on how people with lived experience of OCD and clinicians felt VR could be used to model typical behavior and thought patterns to clients with OCD whose experience may mean they are unsure what this looks like. C1 highlighted that some clients do not know how to behave in the absence of OCD due to the prolonged course of their symptoms and the isolation from others. Thus, VR could provide a visual demonstration of a character modeling behaviors (eg, having a shower) in a typical or antiobsessional way. P1 suggested this could also be applied in a new way beyond the physical demonstration to visually model typical mental processes and highlight how intrusive thoughts appear for everyone, but that the interpretations of those thoughts differ between people with and without OCD:

I was trying to work out how you could really show the person who’s doing the VR that the person who’s in the VR doing the activity wasn’t having the same thoughts, but so I was thinking like word bubbles, but then also kind of possibly like heart rate down the side of just like average whereas you know like I would know mine would be going 300 beats a minute and theirs is just like 60.

[P1]

Clinicians and people with lived experience of OCD felt that this modeling strategy could be augmented by having the character they watch in the VR actually be a version of themselves, building upon the idea of the feedforward mechanic shown in the educational presentation at the end of Workshop 1 [41]. Both groups felt this feedforward idea could be adapted from its use in an athletic context in the presentation into a form of “self”-modeling that could act as a form of rehearsal for the client or act as an approach supporting behavior, something which encourages clients to attempt to engage in the exposure themselves. People with lived experience of OCD felt this could help support generalizing learning to different situations as well, as the avatar of the client could be shown doing exposure exercises in many different environments.

### Theme 3: Illustrating Metaphors and the Treatment Model

#### Overview

This theme reflects how clinicians and people with lived experience of OCD felt VR’s visualization capabilities could be used for the purpose of better integrating metaphor into treatment and supporting clients’ application of the cognitive model to their treatment experience. Metaphor was frequently mentioned in both Workshop 1 and 2 as a technique therapists use to help clients understand and address their OCD, and VR could act as a more impactful delivery system for those metaphors. These ideas are delineated into 2 subthemes: the first is externalizing and visually representing OCD as a metaphorical character in the VR with the client—this has some research support already in the field of anxiety disorders, so stakeholders’ ideas may be an adaptation of this [47]. The second is using VR to support clients’ understanding of the treatment model and actively apply it throughout their treatment using novel context-relevant cues to keep the model present in clients’ minds.

#### Representing an Externalized OCD

Clinicians felt that rendering OCD as a visual entity in VR separate from the client could help better demonstrate the cognitive processes (eg, lack of disconfirmation, the effects of safety-seeking behaviors, and neutralizing) at play in the maintenance and treatment of OCD. This could be done as a stand-alone activity where the therapist explains treatment concepts with a metaphor for OCD while the patient watches a visualization of this metaphor in VR. For example, OCD is sometimes explained to clients with the “Anxiety Monster” metaphor. This metaphor represents the process by which OCD begins as an innocuous response to normal anxiety, but over time, as the client interprets it as a threat and seeks safety rather than confronting it, it becomes a more dominant force that grows and controls them more.

Alternatively, clinicians suggested having the externalized OCD be diegetically present during VR exposure work and reacting to the client’s actions to reinforce the learning clients should take from the exposure. For example, if a client successfully carried out an exposure without responding, the OCD representation might shrink in size, or express upset and metaphorically “lose power,” which would be shown to the client in real time.

When you start starving that monster, when you start saying no, the monster starts losing weight, getting smaller and so eventually his voice is so small it no longer controls you. So you can do some kind of virtual reality imagery around this […] It’s all about the person practically playing out saying “no, I’m not gonna give you that.”

[C3]

There was significant discussion from both clinicians and people with lived experience of OCD over the most appropriate form for the externalized OCD to take and the nuances of its design. For example, clinicians highlighted The Anxiety Monster and The OCD Bully as 2 metaphors they regularly used in their current treatment of OCD to illustrate the effect of counterproductive strategies such as safety-seeking behaviors on the maintenance of OCD. However, some felt that a visualized OCD Bully might be too visceral an image as many people have previously experienced bullying. Additionally, some felt that any sort of Anxiety Monster should not be too “evil” but rather “annoying,” having developed from a relatively harmless symbol of anxiety. Conversely, P7 felt that the idea of OCD as a “monster” that shrinks was not helpful, as it did not effectively acknowledge that the origin of the thoughts manifesting as OCD has come from a place of normal anxiety that at one point acted as a form of self-preservation.

I kind of always see it as, you know, if you’re challenging these things, this thing grows because it’s not as frightened anymore, you know, rather than it be a scary, horrible monster, which I know will probably work for a lot of people, I prefer the visualization where things grow the less you do it because you’re enriching it.

[P7]

Similar to this idea, C1 suggested visualizing an externalized “self”; an image of the client that exists in the VR alongside a representation of the OCD. When the client is doing an exposure, their virtual “self” supports and reminds them why they are doing exercises they find difficult to tackle OCD, and as compulsions are resisted, their virtual “self” grows while the OCD representation shrinks.

Additionally, people with lived experience of OCD suggested that externalizing OCD might help make it feel more contained and manageable when addressing it in treatment, although this is likely to differ depending on the individual and demonstrates the importance of accounting for individual preferences. Both groups suggested it might help clients in thinking about cognitive aspects of the treatment such as Theory A and Theory B during exposure. The externalized OCD might support the process of examining their thoughts and interpretations during exposure and highlight “OCD interpretations” of intrusive thoughts.

#### Supporting the Application of the Cognitive Model During Treatment

Clinicians and people with lived experience of OCD felt that several aspects of the cognitive treatment model could be supported with the use of VR. Given the importance of understanding and applying the treatment model to everything clients do, VR could effectively provide this in a variety of ways.

The predominant method suggested to help clients apply the model to their work in therapy was to provide prompts during VR exposure exercises encouraging reflection on their cognitions. The prompts could be simple pop-up reminders, either as text like “It’s the way you’re responding to the thought, not the thought that’s the problem” (P5) or diegetically embedded in the scenario, for example, a character in the scenario talks to the client. However, clinicians felt that an interactive system active during clients’ exposure exercises would support them in actively reflecting on their experience and applying it to their learning about their cognitions and reduce the potential for them to just passively experience and endure the exposure. The VR app could periodically interject prompts in the form of questions for the clients that they can respond to as they are doing the exercise about how they are feeling and behaving.

So if the programme is saying “are you doing anything to keep yourself safe in any way?” for the person to then be able to say, “well, actually yes, I’m avoiding looking actually at the child, I’m looking slightly next to it.” So then that then prompting something about also “what do you need to do now? You need to get closer” or so that there’s, it’s much more of a sort of two way interaction and for them to be able to say I am doing this, theory B would tell me that and this is what I'm learning, not just to be told what you're learning.

[C6]

Clinicians noted that this process would require the supervision of a therapist to monitor how the client was reacting to the exposure in terms of anxiety levels and reassurance or avoidance strategies, as these can be difficult for the client to note themselves. These prompts could be random or linked to biofeedback, with the former encouraging consistent application of the model and the latter ensuring clients do not forget to apply the model when overwhelmed.

C5 suggested using VR as a tool for metacognitive work as part of the more cognitive aspects of the treatment model. Metacognitive work involves exercises designed to challenge clients’ currently held maladaptive beliefs about how the world works, for example, an inflated sense of responsibility for adverse outcomes or issues of magical thinking. They suggested VR’s ability to facilitate impossible situations could be used in a similar fashion to the “script-writing” technique. This exercise is designed to translate the client’s beliefs and thoughts into a medium that allows them to then be examined more closely and objectively; for example, the client writes about their OCD as if they were writing a script for a movie. This allows cognitions and beliefs to be examined, questioned, and played with by the therapist and client together to challenge their logic and reduce their power over the client.

It could be like […] “OK, alright, here’s an avatar of my brother, how should he die today? Meteor, or maybe eaten by a dragon […] let’s have a choice” and something like that then, rather than treating the thoughts as really serious and something you’ve got to deal with is like the most important thing actually just going, “it’s just a shitty thought that you don’t have to pay any attention to” and in fact, let’s mess about with the thought.”

[C5]

### Theme 4: “Anxiety Management”: Facilitating Approach and Building a Toolkit

#### Overview

This theme was primarily discussed by people with lived experience of OCD and showcases ideas not considered by the researchers or clinicians about how VR could be used as a tool to help clients manage the extreme anxiety they feel during treatment in ways conducive to their treatment. The intention behind this would be to support continued engagement in treatment and can be explained in the 2 subthemes. First, to use VR as an “anxiety management” tool within exposure exercises to support clients through feelings of overwhelming anxiety that might prevent them from engaging fully in the exposure. The second refers to the use of “anxiety management” tools and techniques outside of exposure work as part of a wider toolkit clients can use alongside their CBT.

#### When Overwhelmed During Exposure

Extreme anxiety is commonly experienced in exposure exercises. People with lived experience of OCD suggested that VR could be used to support clients if that anxiety ever became extreme to the point of being overwhelming and preventing them from engaging in the exercise properly. They proposed using motivational prompts (such as voice messages or images from loved ones) or having access to a virtual “safe space” to help manage anxiety and allow continued effective engagement in the exposure. It was raised that these tools could be used both to complement in vivo exposure as well as part of a VR exposure.

When I’m in the middle of an exposure and my anxiety’s sky high […] I totally forget what to do, so it might be helpful to have like prompts saying ‘do you want to see a face of a loved one? Do you want to go to your safe place?’ and then you just press a button to do that. Because […] I just freeze.

[P1]

However, people with lived experience of OCD acknowledged that the use of these tools could constitute avoidance strategies if not implemented properly and perhaps hamper progress.

There’s two aspects to it, isn’t there? Like if, if you were doing that to remind yourself why you were continuing with the exposure, I think that would be like in line with ACT, but then if you were doing it to reassure yourself and to avoid the exposure, then you’d be negating what you've done […] so it’s very important to like to do it in the ACT way rather than the avoidance way.

[P4]

#### Building a Toolkit for Anxiety Outside Exposure

Primarily, people with lived experience of OCD felt that VR could provide clients with tools they could use outside exposures; both to ground them immediately afterward, and to develop anxiety management skills for wider recovery.

People with lived experience of OCD suggested several examples of tools that could be used in both instances, including “safe” or meditation spaces (optionally cocreated with the patient), guided imagery, grounding exercises (such as the 5-4-3-2-1 anxiety coping exercise), or even readily available VR games. For immediate postexposure purposes, there was an emphasis on these activities being quick and guided, due to the energy it takes to do exposure exercises. It could even automatically move the client from exposure to grounding, removing the impetus from the client to come up with a strategy after a difficult session.

I think there should also be the option to be to be able to access it away from exposure time, because that’s a skill, isn’t it, mindfulness and grounding. So if you had the option to visit those techniques and practise them at other times, even when it’s not exposure time, I guess you'll get better at them?

[P7]

Building on the idea of a “safe space,” C1 proposed externalizing a representation of the client’s self-image as an avatar in the space called a “compassionate self.” The client could customize this “compassionate self” however they wanted, and it would then walk them through the postexposure routine a therapist would normally do. C1 felt this could give clients a powerful image of self-compassion to hold onto when not in the therapy room.

### Theme 5: Motivation

Clinicians and people with lived experience of OCD felt that VR could be used as a motivational tool by demonstrating to clients what their lives could look like when OCD disrupts them less severely. They proposed this might help clients engage in the difficult parts of therapy by making their goals more consistently apparent and reminding them why the difficulty of treatment is worth it.

One proposed mechanism for this (both within and between sessions) was to immerse people in environments and scenarios that they aspired to engage in after therapy. Both groups felt this could help clients build goals and reclaim parts of their life they felt were “lost” due to OCD. A clinician even suggested an “unlocking” mechanic, where the client symbolically “unlocked” and reclaimed parts of their life by successfully completing exposure exercises relevant to their goals.

You know, we obviously get people to set goals and think of an imagined future that is better than the one that they have, but we could capitalize more on that. Potentially you could have non-OCD land; you could just go and visit that.

[C2]

Both groups also discussed the idea of visualizing clients’ progress and therapy journey. Clinicians felt this could act as a good motivational tool if clients could predict how they would fare in exercises and then watch their actual performance back. People with lived experience of OCD additionally felt this might help clients break their treatment down into understandable steps of how they will address their OCD. However, they also highlighted the potential for this to backfire and affect self-esteem if clients felt they were not achieving their goals.

Alternatively, similar to the “self”-modeling mentioned in Theme 3, clinicians and people with lived experience of OCD proposed using an avatar of the client as a motivational tool. This was also likely inspired by the presentation of the feedforward mechanic [41] at the end of Workshop 1, but adapted to the context of motivation to engage in treatment for OCD. One idea was to have this avatar demonstrate behaviors the client aspired to do (in a highly personalized format for increased emotional weight) in a way they currently felt unable to with their OCD, perhaps even with visual thought bubbles demonstrating their mental processes as well. Another was to have the avatar be diegetically present during exposure exercises, either as a visual reminder in the background of the client’s goals, or alternatively as a supportive figure giving the client direct encouragement.

Is she able to kind of just watch herself going around and making food without any repetitive motions or anything like that, you know, and maybe her son is just sitting there coloring and he’s not given up waiting for dinner because it takes her forever or something like that?

[P7]

Finally, P7 suggested gamification of treatment content may be useful for some people. For example, they felt that competing against your past performances in an exposure exercise could be quite a good motivator to push harder and could be a reward for some people. However, they expressed the possibility that “losing” could damage confidence and motivation, as well as the fact that gamification likely would not universally motivate everyone.

## Discussion

### Principal Findings

This study aimed to generate novel ideas for the application of VR to the treatment of OCD from relevant stakeholders who might benefit from the use of such ideas in treatment. To achieve this, we took a 2-phased approach using participatory design workshops to first understand stakeholder perceptions of the current state of OCD treatment, then, second, generate ideas for novel applications of VR to support treatment. The PBA for intervention development [30] posits that the generation of new ideas should be rooted in an understanding of the context of the issue. By discussing the current state of treatment with stakeholders in the first phase of workshops, this context was developed and shared between all researchers and participants. This then supported the generation of some new ideas related to VR and the refinement and application of others to the context of OCD in the second phase of the workshops.

The first phase of workshops investigated stakeholder views of the current state of OCD treatment and identified several important features of successful OCD treatment. These themes were (1) developing a shared understanding of the model, (2) quality exposure work and behavioral experiments, and (3) therapeutic alliance. The findings from Workshop 1 are largely in line with previous literature about high-quality treatment for OCD [44-46]. Of particular note from the workshops was the emphasis on the need for the client to understand how the CBT treatment model works and to buy into its effectiveness, and for treatment to be adaptable and personally relevant to clients.

Workshop 1 provided researchers and participants with a contextual understanding of the perceived current state of OCD treatment, including key features to keep and areas to develop upon. Workshop 2 produced a wide range of ideas for how VR could support the treatment of OCD. These could be delineated into 5 themes based on the proposed mechanisms for how they would support treatment: (1) enabling exposure work, (2) modeling, (3) illustrating the treatment model, (4) “anxiety management,” and (5) motivation. It is worth noting that the novelty of the ideas proposed in Workshop 2 varies. For example, the idea of a self-modeling avatar is likely not totally organic, but based around the idea of feedforward presented as an example at the end of Workshop 1. It could be argued that this presentation might have primed workshop participants to consider this particular idea for development. Additionally, the idea of enabling exposure exercises for impossible scenarios is not entirely novel, but rather an extension of previous VR research in OCD where it is used to provide access to normally inaccessible situations [18,21]. As such, the claim that all the ideas generated from Workshop 2 are entirely novel should be tempered with the understanding that some of these ideas likely have origins in the participants’ background and the researcher’s influence.

Nonetheless, the findings from Workshop 2 suggest that there is a much broader scope for the application of VR to the particular context of the treatment of OCD than has previously been explored [25]. While both stakeholder groups contributed to all themes, ideas from clinicians predominantly centered around how VR could support consistent and high-quality application of the cognitive model. Perhaps expectedly, there were several ideas involving VR in exposure work, for example in enabling exposures for scenarios which are normally impossible to facilitate and thus helping ensure the quality of exposure conducted in CBT [16,17]. However, despite previous studies proposing VR simply as an effective one-for-one in vivo replacement [25,47,48], the majority of ideas presented in Workshop 2 went beyond this. For example, by making cognitive concepts of the model accessible to clients and interactive during exposure work through integrated real-time metaphorical and theoretical framework prompts and self-modeling tools. Several of the proposed ideas already have some research support in other areas, so are not entirely novel but have been adapted to the field of OCD by the stakeholders. For example, several participants proposed adapting the idea of feedforward for athletics training [41] that was presented at the end of the first workshop into a self-model for exposure exercises. Additionally, some suggested the idea of the creation and destruction of an anxiety avatar, which has seen some interest already in other mental health disorders such as anxiety [47]. It is possible that the use of VR can help clients engage in metaphors in a more tangible, multisensory way, thus making it easier to continue applying and learning from the cognitive model [49]. VR could also augment the cognitive element of CBT through visualized metacognitive strategies to address clients’ thought processes more directly. Recent reviews suggest that metacognitive therapy using methods like the ones described in the “Supporting the Application of the Cognitive Model During Treatment” section may be as effective as gold-standard ERP in reducing OCD symptoms [50]. As discussed in the “Results” section, VR could support the administration of these exercises by allowing visualization and interaction with these exercises and concepts, highlighting a completely untapped avenue for VR to enhance treatment.

People with lived experience of OCD made suggestions that aligned with those of clinicians; however, they predominantly focused on using VR to support engagement with treatment. This was proposed through the use of “anxiety management” tools to prevent overwhelming anxiety and disengaging from effective exposure work, and through motivational measures to support engagement with the treatment as a whole. The purpose of exposure exercises and behavioral experiments is to elicit similar feelings to those clients experience in situations they find difficult in their daily lives. By confronting these feelings and reflecting on the cognitive model, they can change their beliefs and behavior around them [51]. The focus of people with lived experience of OCD on these ideas surrounding “anxiety management” suggests that there may be value in investigating VR’s ability to address this overwhelming anxiety as part of a treatment protocol for OCD. This idea is hitherto uninvestigated in the field of VR for OCD; however, there has been some evidence of success with this idea in generalized anxiety disorder [52,53]. The primary mechanism of these instances is the idea of a relaxing “safe” space, so the other ideas mentioned by stakeholders, which go beyond this, may provide plentiful opportunities for further research.

### Limitations

Several limitations of the study should be considered when interpreting these findings. First, the stakeholder sample was relatively small and homogenous. Both the clinicians and people with lived experience of OCD who participated in the study were recruited from established OCD research and involvement groups, or from the clinical supervisor’s contact networks. Consequently, clinicians were highly experienced in treating OCD, while people with lived experience of OCD had previous experience participating in OCD-related research and were comfortable discussing their experiences in group settings. The findings may therefore reflect the perspectives of a particularly engaged and research-active group of stakeholders and may not fully represent the views of individuals with OCD who have had less contact with treatment, research, or coproduction activities. This is relevant to bear in mind, as several ideas proposed in the workshops saw immediate disagreement between stakeholders, so it is possible that people with lived experience of OCD who are not comfortable engaging in research or treatment might object to other proposed ideas. Additionally, all participants identified as White British, and approximately two-thirds of participants in both stakeholder groups were female. Given evidence that OCD presentation, help-seeking behaviors, and treatment experiences can vary across demographic groups [54-56], alternative priorities and perspectives may have emerged from a more diverse sample.

Second, the small size of the sample in the study may raise concerns over whether the array of ideas in this study can be considered exhaustive. The study was exploratory in nature, with a focus on generating a breadth of stakeholder-informed ideas for future intervention development, rather than providing a comprehensive account of stakeholder experiences. Data saturation was therefore not specified as a stopping criterion and was not formally assessed. The CQA approach taken in this study is based on thematic analysis, where there has been some debate over the utility of trying to achieve data saturation [57]. While a broad range of perspectives and design ideas were generated across workshops, additional stakeholders may have contributed further ideas or refinements. The findings should therefore be viewed as an initial set of stakeholder-informed design concepts rather than an exhaustive account of all potential applications of VR within OCD treatment. Logistical constraints meant that Workshop 2 sessions with participants were often limited to 60‐ to 90-minute meetings, which limited the development of some of the ideas. Ideally, according to the PBA [30], there would be a process of idea refinement following the initial generation of ideas. Due to the time constraints of the project, we could not engage in this stage of the approach. As such, while many ideas from Workshop 2 contain sufficient detail to begin prototyping, there are some which lack detail on how they might specifically be implemented or how they would work in practice. In these cases, the ideas are better categorized as design concepts rather than fully formed methods ready for development and implementation. However, given the primary aim of the workshops was to generate novel ideas, the goal has still been achieved, albeit with some areas for elaboration left outstanding. Future researchers could continue the process of iterative development with more design workshops and larger, more diverse samples. However, these could be extended to include iterations focused on refining existing concepts to translate more of these ideas into actionable development prospects.

In a similar vein, the thematic structure of the analysis could be argued to be a limitation from the perspective of clinical psychologists looking to develop specific interventions from the ideas in this study. While this study gives readers an overview of the potential for VR to support treatment, it does not provide explicit, fully refined ideas that can be taken into further development. For example, the idea of the “self” avatar spans several different themes, including modeling, motivation, and “anxiety management,” and there is no single description of how to implement it into treatment. Researchers seeking to test and implement a specific idea would need to first carefully check each theme and subtheme of the analysis to understand the proposed mechanisms through which that idea could support treatment. Further development of any of the ideas contained in this study should involve further elaboration and consultation with relevant stakeholders in the design process.

### Implications for Future Research

The major implication of this study for future research is that it provides a wealth of stakeholder-supported novel VR methods which could be developed and applied to support or improve the implementation of CBT for OCD. Furthermore, due to the participatory design nature of the study, these methods are targeted toward addressing issues raised by people with the most experience of OCD who best understand the difficulties of its treatment. As such, this workshop study provides several potential treatment tool mechanisms for other researchers to develop and investigate. For example, enabling the conduct of exposure exercises which are normally impossible and augmenting the learning obtained from exposure exercises by more directly including aspects of the cognitive treatment model seem to be the most endorsed and well-developed concepts from the workshops. These are also the most geared toward supporting the high-quality administration of the cognitive treatment model. However, there was also significant support for ideas focused less directly on the model, such as visualizing client goals and supporting the management of anxiety during exposure exercises to maintain effective engagement. Further participatory design or development projects could select, expand, and refine ideas raised in these workshops to produce applications supported by stakeholder design throughout the process.

The second avenue for future investigation would be to run similar workshops with other groups of clinicians and people with lived experience of OCD to include a greater breadth of experiences in design ideation. As was raised several times throughout the workshops, OCD can be a highly idiosyncratic experience for individuals, and some ideas that were raised were not seen as universally applicable. Indeed, there is evidence that cultural and social differences between people affect experiences of using services for OCD [56]. Replications of this study with different ethnic and social groups could help understand how the ideas proposed in this workshop would be perceived in different care settings.

This study demonstrates the power of participatory design in applying VR to the treatment of OCD. With the ideas raised by participants, there are a large number of avenues and novel methods for researchers to test in applying VR to support and enhance the treatment of OCD. It is envisaged that refining and applying these ideas, the theoretically effective treatments already available might be more consistently applied at a high quality, with support from such VR apps.

## Supplementary material

10.2196/92410 Multimedia Appendix 1 Additional prompts and follow-up questions used in Workshop 1.

10.2196/92410 Multimedia Appendix 2 Brief educational presentation on virtual reality.

10.2196/92410 Multimedia Appendix 3 Persona development worksheet.

## Abbreviations

CBT: cognitive behavioral therapy
CQA: collaborative qualitative analysis
ERP: exposure and response prevention
JARS-Qual: Journal Article Reporting Standards for Qualitative Primary Research in Psychology
MRC: Medical Research Council
OCD: obsessive-compulsive disorder
PBA: person-based approach
RQ: research question
VE: virtual environment
VR: virtual reality
