# Supplementary material for: Novel Virtual Reality Methods for the Treatment of Obsessive-Compulsive Disorder Using a User-Centered Design Approach: Focus Group Study
Source: JMIR XR Spat Comput. 2026 Aug 20;3:e92410. doi: 10.2196/92410 (PMC13494860; doi:10.2196/92410)
Supplement: Supplementary file 1 [file xr-v3-e92410-s001.docx]

## **Multimedia Appendix 1: Additional Prompts and Follow-Up Questions Used in Workshop 1**

**Table 1.** Additional prompts and follow-up questions for Workshop 1 – focus group discussions

| **“Past experiences of receiving/providing treatment for OCD”** | **“Improving the treatment of OCD”** |
| --- | --- |
| Did you find treatment effective in addressing symptoms?  What about it was effective in terms of treating symptoms?  What was less effective in terms of treating symptoms?  Were there any things that seemed pointless, or that actively made things worse e.g., specific exposure exercises, homework assignments?  Can you pick out a single exercise or aspect that you think helped you the most?  Are there more general things that helped treatment be effective – things like the atmosphere of the clinician’s office?  Have you used technology at any time in your treatment? How did you find it? Which client group found it most useful?  Were there things that made clients feel engaged in therapy?  What were they?  Were there specific exercises, homework assignments, client interactions?  What didn’t work well in terms of engagement in therapy?  What were the hardest parts of therapy for your clients?  Were there particular exercises that were difficult to engage with? | Think of a time in treatment that was difficult for you (or your client) – what would you have changed?  What would you have wanted to happen differently?  What would have made the difficult parts of treatment easier?  Think of a time in treatment that did not feel helpful – or that felt ineffective. What would you have changed for it to have been more helpful for you?  Was there anything you felt was totally missing from your treatment? |
