# Supplementary material for: Novel Virtual Reality Methods for the Treatment of Obsessive-Compulsive Disorder Using a User-Centered Design Approach: Focus Group Study
Source: JMIR XR Spat Comput. 2026 Aug 20;3:e92410. doi: 10.2196/92410 (PMC13494860; doi:10.2196/92410)
Supplement: Supplementary file 2 [file xr-v3-e92410-s002.docx]

## **Multimedia Appendix 2: Brief Educational Presentation on VR**

*(Video showcasing two different iterations of OxfordVR’s acrophobia VR treatment tool)*

To finish off today I wanted to give a quick primer ahead of our next workshop. Like I said, VR has already been used in a couple of settings in psychological treatment. One example I like comes from OxfordVR, who created a VR application to help treat fear of heights.

As you can see people are put into a shopping mall-like environment, which has multiple levels of different heights. In the application, an automated virtual therapist talks them through various exercises, designed to expose them to heights and help them acclimatize to their responses.

I like this application as an example of VR in therapeutic settings, because you can see the ways they start to explore VR’s full capabilities while seeking to provide good treatment outcomes. For example, I think the shopping mall is quite engaging in the way it’s strikingly designed with those fantastical elements like the flying whale or the spheres floating around, but these elements are also quite effective reminders for the client of the space they’re in, and the perception of height. I don’t know how much this contributes to their treatment outcomes, but I think it’s interesting to see them pushing beyond replicating the real world and exploring things beyond reality.

I recently did a systematic review of all studies I could find that involved using VR in OCD in some way. So far there is promising initial evidence that VR is effective for exposure in terms of evoking OCD symptoms, and it shows signs of being able to reduce anxiety and OCD symptoms with repeated use, but this isn’t as well-supported.

Like I’ve said before, all the VR applications I found work by simply replicating real-life exposure scenarios. These scenarios are all focused on contamination-related OCD, and it SEEMS as if VR would be most helpful with these symptom dimensions, however, I don’t want to discount anything at this stage and want to keep an open mind where possible.

Obviously, we’ve been talking about all of this in the context of exposure, but again, while it SEEMS as if this treatment mechanic is where VR is most applicable, I don’t want to discount ideas about other ways of using VR. Because there’s some potentially pretty out-there things you can do with VR, and I’m open to all ideas of how they can be applied to treatment…

*(Video showing the use of a feedforward mechanic in a VR exergame to increase intrinsic motivation to exercise ^40^)*

For example, one interesting thing you can do with VR is called feedforward – this has been applied in sports, but not yet in psychological treatment. The idea of feedforward is that we learn from our **future** selves, rather than our past selves, as is the case with feed**back**. By watching an avatar of ourselves do something in an improved way from what we can currently do, this supposedly helps improve our performance – this video demonstrates this concept in the context of cycling.

A researcher here at the university creates an avatar of himself and then programs that avatar to cycle just slightly faster than he can at the moment. The idea then is that he races against this improved-self avatar, which pushes him to improve his performance proactively rather, than reflecting on past sprint times, for example.

Like I said, I don’t know if this is applicable to a treatment context, but I hope it demonstrates the idea that things are possible in VR, that are not in the real world. And I want us to explore whether this fact can be harnessed for our purposes.

Of course, that’s just one example of how we might use VR in novel ways to enhance treatment. You could potentially argue, from the OxfordVR examples, another way we could use VR is the gamification of some exposure exercises, as with some of those in the fear of heights application, which might encourage engagement in exposure.

And this will be the aim of the next workshop – to get creative about the ways in which we could use VR to treat OCD. I’d encourage you to let this sit in the back of your mind between now and the next workshop and ask yourself the question “If anything was possible, and I as the clinician could do anything, what would I want to happen when I provide treatment?”

Thank you very much for coming today and sharing your thoughts.
