# Supplementary material for: Novel Virtual Reality Methods for the Treatment of Obsessive-Compulsive Disorder Using a User-Centered Design Approach: Focus Group Study
Source: JMIR XR Spat Comput. 2026 Aug 20;3:e92410. doi: 10.2196/92410 (PMC13494860; doi:10.2196/92410)
Supplement: Supplementary file 3 [file xr-v3-e92410-s003.docx]

**Multimedia Appendix 3: Persona Development Worksheet**

| **Their name is:** | **They are:      years old**  **They identify as a**:  **They live in:** |
| --- | --- |
| **Family members (and children):** | |
| **Main responsibilities in life:** | |
| **Hobbies/other interests:** | |
| **Key features of their daily/weekly routine:** | |
| **Intrusive thoughts they deal with:** | |
| **Situations they find difficult:** | |
| **Compulsive behaviors they perform:** | |
| **Other important details about them**: | |
